# Implicit Causality and Pronoun Resolution in Intersubjective Discourse Relations

**DOI:** 10.3389/fpsyg.2022.866103

**Published:** 2022-05-10

**Authors:** Siqi Lyu, Luming Wang

**Affiliations:** ^1^Institute of Psychology, University of Tartu, Tartu, Estonia; ^2^College of Foreign Languages, Zhejiang University of Technology, Hangzhou, China

**Keywords:** implicit causality, pronoun resolution, intersubjectivity, concession, perspective shift

## Abstract

Interpersonal verbs like *disappoint* and *praise* in *Lucy disappointed/praised Mary because she…* bias the potential cause of the event to one of the antecedent noun phrases (henceforth NPs) (e.g., Lucy for *disappoint* whereas Mary for *praise*). Using Chinese as its materials, this study investigated how verb-based implicit causality affects online pronoun resolution in backward concession (e.g., *Lucy disappointed/praised Mary although she…*), an intersubjective discourse relation where the subordinate *although*-clause forms an indirect relationship with the preceding main clause. Experiment 1 was a baseline experiment with the typical structure where implicit causality is found to be effective, i.e., backward causality. Results showed a clear modulation effect of implicit causality on pronoun resolution such that as verb bias strength decreases, participants were faster in processing sentences that disambiguate the pronoun to the verb-inconsistent NP. However, this modulation effect was not observed in Experiment 2 where we used the same verbs but replaced *because* with *although*. There was no preference for the pronoun to be disambiguated toward the verb-consistent NP or the verb-inconsistent NP in backward concession. The results of Experiments 1 and 2 were replicated in Experiment 3 where we directly compared causal and concessive relations. We suggest that the absent effect of verb-based implicit causality in backward concession could be attributed to the intersubjective nature of the concessive relation. Discourse devices such as *although* indicate speakers’ subjective perspective and comprehenders are able to quickly accommodate the speaker’s point of view during online discourse processing.

## Introduction

Successful communication relies on the correct interpretation of pronouns like *it, he/she*, and *they* in context. Comprehenders make use of various sources of information in discourse to retrieve the correct referent of pronouns (Kehler, 2002). Certain interpersonal verbs have statistically reliable preferences about their causal antecedents (“Implicit Causality”; Garvey and Caramazza, 1974). For example, interpersonal verbs like *disappoint* in (1a) and *praise* in (1b) have an implicit feature of attributing the potential cause of the event to the first noun phrase (NP1) [e.g., *Lucy* in (1a)] or the second noun phrase (NP2) [e.g., *Mary* in (1b)], respectively. Whereas it is likely that Mary was disappointed because of something Lucy did, there is a preference that the reason why Lucy praised Mary is because of something Mary has done.

(1) (a) Lucy_*i*_ disappointed Mary because she_*i*_… (NP1-biased verb).
(b) Lucy praised Mary_*i*_ because she_*i*_… (NP2-biased verb).

This implicit feature of interpersonal verbs has been observed in other discourse relations than causality. For instance, an opposite pattern is observed in sentences that describe the potential consequence of an event with a subordinate *so*-clause (‘Implicit Consequentiality’; see Au, 1986; Stewart et al., 1998; Cheng and Almor, 2017; Garnham et al., 2021; among others). Whereas the pronoun in *Lucy disappointed Mary so she…* is most likely to refer to Mary, that in *Lucy praised Mary so she…* tends to refer to Lucy. In another study, Xu et al. (2019) examined pronoun resolution in different discourse relations and found a distinctive feature of concessive relation such that compared with other relations like *so-*clause, *and-*clause, or two independent clauses (without any connectives in between), *although*-clause was processed with greater uncertainty.

Concession has been considered as a negated causal relation (König, 1991; König and Siemund, 2000; Louwerse, 2001) but differs from causality in that it involves the speaker’s subjective attitude (Verhagen, 2005; Lyu et al., 2020). The aim of the present study is to investigate how verb-based implicit causality affects pronoun resolution in backward concession, an intersubjective discourse relation where the subordinate concessive clause (henceforth C2) forms an indirect relationship with the preceding main clause (henceforth C1). Below we first introduce subjectivity and intersubjectivity on theoretical accounts, and then move to previous findings on the influence of verb-based implicit causality on online pronoun resolution, followed by a review of previous experiments on the processing of concession in discourse and an overview of experiments in the current study.

### Subjectivity, Intersubjectivity, and Concession: Theoretical Accounts

A coherent discourse consists of various types of relations between events. For instance, (2a) describes a causal relation where the causal event *she worked hard (p)* and its consequence *she passed the oral English test (q)* form a typical cause-consequence relationship *p → q* in the real world. On the other hand, what is described in (2b) concerns the speaker’s reasoning such that the observation that *she passed the oral English test (p)* is an argument for the claim that *she worked hard (q)*. Relations like (2a) are considered as an objective relation and those like (2b) subjective, respectively (Sanders and Spooren, 2015; Kleijn et al., 2021; see also “content domain” and “epistemic domain”, respectively, by Sweetser, 1990).

(2) (a) She passed the oral English test today, because she worked hard. (content domain)
(b) She worked hard, because she passed the oral English test today. (epistemic domain)

Concession has always been considered as a negated causal relation (König, 1991; König and Siemund, 2000; Louwerse, 2001). Whereas the two events *work hard (p)* and *pass the oral English test (q)* in (2a) form a causal relation *p → q* as indicated by the connective *because*, the causal link between events is rejected in a concessive relation like (3a), where the concessive connective *although* leads to a negated consequential event *fail the oral English test (q)*. Similarly, whereas (2b) describes an epistemic causal relation between *passed the oral English test (p)* and *work hard (q)*, a concessive relation like (3b) claims a negated conclusion, i.e., *she didn’t work hard (q)*.

(3) (a) She failed the oral English test today, although she worked hard. (content domain)
(b) She didn’t work hard, although she passed the English test today. (epistemic domain)

Concession additionally features an *intersubjective* coordination between the speaker and the addressee. A wide range of linguistic phenomena involve “connecting, differentiating, and ‘tailoring’ the contents of points of view with respect to each other (rather than organizing a connection to the world)” (Verhagen, 2005, p. 4), of which concession is one. On this account, the concessive connective in *q although p* evokes two mental spaces (Fauconnier, 1994) with different epistemic stances, one in which the speaker acknowledges a potentially valid inference *p → q*, and the other in which the speaker displays a negative epistemic stance toward the proposition *p* herself. In saying a concessive sentence like (3a), for instance, while the speaker acknowledges that given *p* (i.e., she *worked hard*) there may be good reasons to adopt *q* (i.e., *she passed the oral English test*), she nevertheless invites the addressee to adopt a contradictory result, i.e., *she failed*, which is incompatible with *q*. Even though a concessive relation like (3a) describes events in the content domain that is usually considered objective, it comes with an inherent *intersubjective* feature that the speaker invites her addressee to accept a contradictory conclusion.

In some cases, *although* doesn’t express a real-world relationship between two states of affairs in the way *because* does (Iten, 2005, p. 163). Take (4) as an example, where the two clauses are relatively independent from each other and do not necessarily form a (negated) cause-consequence relationship. The two clauses connected by *although* in (4) are not based on the real-world knowledge that *if one lost a flight ticket (p) then she will not fail the oral English test (q)*, which is not a plausible logic in the real world. Rather, (4) is likely to be interpreted as a plausible sentence such that the first clause is a claim made by the speaker and the second is a retreating statement to it. In such cases, the two events are not directly related. The speaker adds some unrelated information that she wants the addressee to accept despite the fact that she acknowledges in the first clause, and the addressee is very likely to tolerate the sentence and accept whatever the speaker provides.

(4) She failed the oral English test today, although she lost a flight ticket last weekend.

A backward causal relation, however, expresses a “tighter” cause-consequence relationship between two clauses. In (5), for instance, the causal connective *because* cues readers of a real-world cause for the event described in the first clause. In this case, comprehenders will try to build up a cause-consequence relationship *if one lost a flight ticket (p) then she will likely pass the oral English test (q)* when reading (5). However, given that such a cause-consequence relationship is illogical in the real world, the sentence is likely to be considered implausible.

(5) *She passed the oral English test today, because she lost a flight ticket last weekend.

As Verhagen (2005, p. 170) puts it, backward concession can “easily produce an effect of retreating and weakening the original claim.” Different from a backward causal relation, backward concession is by default an intersubjective relation. The two clauses of backward concession form an indirect relationship where the concessive clause provides the speaker’s retreating statement to the proposition claimed in the preceding main clause.

### Implicit Causality and Pronoun Resolution

When describing the relationship between two entities in structures like *NP1 V NP2 because…*, the interpersonal verb biases the potential cause of the event toward one of the antecedent NPs (Garvey and Caramazza, 1974). Take (1) as an example, repeated here as (6). There tends to be an overall preference for comprehenders to consider *Lucy* as the underlying cause of the event of *disappointing* in (6a). By contrast, in (6b), readers will tend to consider *Mary* as the implicit cause of the event of *praising*. When verb’s bias is defined as the proportion of completions in which the first noun is the antecedent, bias values range continuously from 1.0 (NP1 is the unanimous antecedent) to 0.0 (NP2 is the unanimous antecedent) (Caramazza et al., 1977). The verb implicit causality can thus be presented as a continuum, to the one end standing the NP1-biased verbs like *disappoint* in (6a), and to the other end the NP2-biased verbs like *praise* in (6b).

(6) (a) Lucy_*i*_ disappointed Mary because she_*i*_… (NP1-biased verb).
(b) Lucy praised Mary_*i*_ because she_*i*_… (NP2-biased verb).

Verb bias is not an absolute constraint on the interpretation of the pronoun (Koornneef and van Berkum, 2006; Hartshorne and Snedeker, 2013). For example, both sentences in (7) are plausible and coherent despite being inconsistent with the verbs’ biases. That is, the referential preference based on the verb bias can be overridden by the disambiguating information in C2 without rendering the sentence ungrammatical or incoherent.

(7) (a) Lucy disappointed Mary_*i*_ because she_*i*_ had a high expectation.
(b) Lucy_*i*_ praised Mary because she_*i*_ was very satisfied.

Studies have consistently shown that comprehenders make use of the information of verb-based implicit causality to resolve the pronoun during real-time processing (e.g., Caramazza et al., 1977; Vonk, 1985; Guerry et al., 2006). In an experiment where participants were asked to read sentences like (8) and indicate their choice for pronoun assignment by saying the appropriate person’s name out loud, Caramazza et al. (1977) found that compared with (8a) in which C2 disambiguated the pronoun to a direction consistent with the verb in C1, participants needed more time to read the entire sentence like (8b) where the verb in C1 and the information in C2 disambiguated the pronoun toward different directions. The results suggest that verbs’ implicit feature of specifying the causal relationship between the participants of an action has an effect on comprehenders’ real-time interpretation of the sentence.

(8) (a) Tom scolded Bill_*i*_ because he_*i*_ was annoying. (verb-consistent condition)
(b) Tom_*i*_ scolded Bill because he_*i*_ was annoyed. (verb-inconsistent condition)

Most of recent psycholinguistic research have focused on when verb-based implicit causality is applied during online processing of pronouns, among which inconclusive results have been drawn with regard to two competing accounts: integration account vs. focusing account. On the one hand, a number of studies found that the information of verb-based implicit causality is only used during sentence-final clausal integration (Garnham et al., 1996; Stewart et al., 2000; Garnham, 2001). For instance, in a series of probe recognition tasks, Garnham et al. (1996) showed that the effect of implicit causality emerges only after the presentation of disambiguating information, i.e., during the integration of the two clauses. On the other hand, more recent studies have adopted the visual-world paradigm and event-related potentials (ERP) and showed that implicit causality was used before the disambiguating information was available (Koornneef and van Berkum, 2006; van Berkum et al., 2007; Cozijn et al., 2011; Järvikivi et al., 2017) or even before participants had encountered the causal connective (Pyykkönen and Järvikivi, 2010). On this account, a sentence fragment like *Lucy praised Mary because…* immediately brings *Mary* into focus at the expense of *Lucy*, and comprehenders prefer to relate a personal pronoun to the most focused antecedent.

Despite the large amount of research on the time course of the implicit causality effect, few studies have zoomed into the subtle difference in the *strength* of verb bias and examine how it interplays with the disambiguating information in C2 in affecting real-time pronoun resolution. As we explained above, implicit causality is a continuum, the two ends of which are the NP1-biased verbs and NP2-biased verbs, respectively (Caramazza et al., 1977). In an offline experiment, Ferstl et al. (2011) systematically measured the implicit causality of 305 English verbs using a sentence completion task and provided the bias score of each verb that ranges from 100 (all completions NP1) to –100 (all completions NP2). As illustrated in (9a) and (9b), *attract* and *abandon* are both NP1-biased verbs but differ in terms of their bias strength. Thus it can be seen that not every NP1-biased verb affects the pronoun resolution to the same extent, and so does the NP2-biased verb.

(9) (a) Lucy_*i*_ attracted Mary because she_*i*_… (bias score 87).
(b) Lucy_*i*_ abandoned Mary because she_*i*_… (bias score 33).

While both the sentence fragments in (9) are likely to be completed with Lucy, as shown in (10), (9a) still has a higher probability than (9b) due to the stronger bias strength of *attract* than *abandon*. From a processing perspective, sentences with strong-bias verb like *attract* in (10a) will likely be easier to process than those with weak-bias verbs like *abandon* in (10b).

(10) (a) Lucy_*i*_ attracted Mary because she_*i*_ was beautiful.
(b) Lucy_*i*_ abandoned Mary because she_*i*_ was annoyed.

It is thus worth taking into account the referential probability of each verb when examining the implicit causality effect. In light of Ferstl et al. (2011), in the present study we will index the strength of the verb bias with the referential probability obtained in a sentence completion task (see section “PRE-TEST”). We will treat the verb-based implicit causality as a continuum and include verbs’ bias scores as a continuous factor in our statistical model, so that a more precise picture of how the pronoun is resolved as the strength of the verb bias changes can be provided.

### Processing Concession in Discourse

Concession has always been studied together with causality and found to be harder to comprehend than causal relations. For example, studies have shown that concessive sentences are processed more slowly than causal sentences, and the recall is worse for concessive sentences than for causal sentences (Townsend, 1983; Caron et al., 1988; Köhne and Demberg, 2012, 2013; Xu et al., 2018; Lyu et al., 2020).

Recently, in a self-paced reading experiment with Chinese materials, Lyu et al. (2020, Experiment 2) found a distinct feature of concession such that although certain “seemingly” implausible words like *kaiche ‘driving’* in (11) induced processing difficulty at the initial stage [i.e., at the critical region, underlined in (11)], it later became as acceptable as the plausible words like *yingyu ‘English’* [i.e., at the post-critical region, e.g., *douzhidao* ‘everybody knows it’ in (11)]. The authors suggest that concession is an (inter)subjective relation that is restricted to the speaker’s cognitive domain and that during online processing, participants can quickly shift their perspective to accommodate the speaker’s point of view and accept the “implausible” content at a later stage.

(11) suiran Ahui yuyantianfu henqiang
although Ahui language talent very good
danshi buxihuan xue yingyu/kaiche
but not like learn English/driving
dajia douzhidao
everybody all know
‘Although Ahui has a talent for language, he doesn’t like learning English/driving. Everybody knows it.’

There are other studies that have demonstrated an indirect relationship between the two clauses in concession. For instance, Townsend and Bever (1982) asked participants to listen to sentence fragments that contain singular morphological bias [e.g., *the boxer want*s in (12)] and then read aloud the interrupting target word (IS or ARE) on the screen as soon as possible. They found no difference in terms of participants’ responses to the singular and the plural target following a concessive fragment led by *though*. The results suggest that the interpretation of incomplete phrases in the later clause of concession is unaffected by the content of an initial concessive clause.

(12) Though the boxer wants to avoid unnecessary injuries, dodging punches… IS/ARE

In another experiment, Morera et al. (2017) presented concessive sentence like (13) in pages (page breaks indicated by “||”). They asked participants to first listen to a fragment concessive clause, after which participants were presented an emotional icon (emoticon) that either matched or did not match the emotional valence of the concessive clause, and then they had to choose the correct continuation of the sentence [indicated in brackets in (13)]. The authors found that the emoticon has no effect on the choice of the correct continuation of a concessive relation, which corroborates Townsend and Bever’s (1978) findings in suggesting that initial concessive clauses open up the possibility for “anything” to follow and are not effective in providing a context with which the subsequent main clause can be integrated.

(13) Although the pupil studied a lot, —— ☺/☹ —— [he passed/he failed] the exam.

In a recent ERP study with backward concession, Xu et al. (2019) showed that resolving the pronoun in a concessive sentence with *although*, as in (14), elicits larger sustained negativity (time-locked to *she*) than sentences containing an explicit consequential connective *so*, a coordinator *and*, or sentences with a full stop between the two clauses. The larger sustained negativity in concession was interpreted as greater uncertainty as to which antecedent would be retrieved as the referent of the pronoun. That is, by the time participants reached *she* in a concessive relation like (14), they were not able to make use of the implicit causality information in the previous context to successfully refer the pronoun to the correct antecedent.

(14) Lily_*i*_ disappointed Nina, although she_*i*_ quit the business. (verb-consistent)

While Xu et al.’s (2019) results showed that the real-time comprehension of the pronoun itself (i.e., by the time participants reached the pronoun) in backward concession was less dependent on the implicit causality information in C1, it remains to be answered whether implicit causality will start to play a role *after* the disambiguating information in C2 has unfolded. If implicit causality in concession plays a role at a later stage (e.g., the integration account; Garnham et al., 1996), then the time taken to integrate C2 *after* it has been completely unfolded should be longer in verb-inconsistent condition than in verb-consistent condition. However, if C1 and C2 in backward concession are relatively independent from each other (i.e., the implicit causality information carried by the verb in C1 has limited effects on pronoun resolution in C2), then there should be no difference in comprehending the verb-consistent and verb-inconsistent conditions even after the completion of the whole sentence. This is the main hypothesis to be tested in the present study.

### Overview of Experiments

The present study aimed at examining how verb-based implicit causality is applied in the online pronoun resolution in an intersubjective discourse relation called backward concession. Experiment 1 tested the validity of the implicit causality effect in Chinese using a structure of [_*C1*_ *NP1 V NP2*] [_*C2*_ *because Pro…*], a typical discourse relation where implicit causality is found to be effective. As a baseline experiment, Experiment 1 only adopted causal relations in the content domain. In a 2 × 2 design, we used both NP1-biased verbs and NP2-biased verbs and manipulated the disambiguating information in C2 as either consistent or inconsistent with the verb bias. Experiment 2 investigated how verb-based implicit causality affects online pronoun resolution in backward concession. We adopted the same design as Experiment 1 but replaced *because* with *although*. In Experiment 3, we crossed discourse relation (causality vs. concession) and consistency of the disambiguating information in C2 (verb-consistent vs. verb-inconsistent) in a within-subject design. By doing so, we are able to directly compare causality and concession and provide further evidence for how implicit causality affects online pronoun resolution in the two closely related discourse relations.

## Pre-Test

In order to obtain the prototypical NP1-biased and NP2-biased verbs in Chinese, a pre-test was conducted in which participants were asked which person Ta “he, she” refers to in sentence fragments like (15). The fragments were created with 150 Chinese implicit causality verbs translated from the English implicit causality verb corpus (Ferstl et al., 2011). All verbs were two-character lexical causatives. The verb was placed between two Chinese nicknames *a-*X ‘‘dear X’’ or *xiao-*X ‘‘little X.’’ Each sentence fragment was followed by three potential answers, including nickname 1, nickname 2, and ‘‘others,’’ presented visually using the Tencent online questionnaire application^1^. Thirty-nine participants (13 men, 26 women, mean age 22.56 years, range 18--35 years) participated in the pre-test and were instructed to choose the best answer that first came to their mind. The referential probability of each verb was calculated, based on which 32 NP1-biased verbs and 32 NP2-biased verbs were selected for the processing experiments^2^. The two types of verbs were matched in terms of their referential probabilities (*p* > 0.2)^3^. The mean referential probability as well as the probability range of the two types of verbs are given in Table 1^4^. See Supplementary Materials for a full list of verbs and their referential probabilities.

(15) A-huang zhenjingle A-hui, yinwei Ta…
A-huang_[*nickname1*]_ surprised A-hui_[*nickname2]*_, because Ta…

**TABLE 1:** Mean referential probability of NP1-biased verbs and NP2-biased verbs.

	NP1-biased verbs	NP2-biased verbs
NP1 (%)	77 (8) [64–92]	18 (6)
NP2 (%)	21 (7)	79 (8) [64–95]
Others (%)	2 (2)	3 (2)

*Means are presented with standard deviations in parentheses. Probability range is shown in square brackets.*

Thirty-two pairs of two-character Chinese names were created and to be used in the experiment as NP1 and NP2, respectively. In order to exclude the potential gender effect brought by the names, in the real experiments we presented critical sentences like (15) in the auditory form, where the Chinese third-person singular pronoun Ta cannot be differentiated in pronunciation (both pronounced as /tā/). Given that in the real experiments, the comprehension questions following each trial with NP1 and NP2 as its potential answers were to be presented visually, we matched NP1 and NP2 in terms of their homophone frequency and number of stokes. The summed homophone frequency and total number of strokes of the two characters in each name were calculated, and separate *t*-tests showed no significant differences between NP1 and NP2 (*p*s > 0.60).

## Experiment 1

Experiment 1 examined the modulation effect of implicit causality information on online pronoun resolution with the typical structure where implicit causality has been found to be effective, i.e., backward causality. We included both NP1-biased verbs and NP2-biased verbs and manipulated the disambiguating information in C2 as either verb-consistent or verb-inconsistent, resulting in a 2 × 2 design.

### Methods

#### Participants

Seventy-five (29 men, 46 women, mean age 19.69 years, range 18–24 years) native speakers of Mandarin Chinese who did not participate in the pre-tests were paid to participate in Experiment 1. Informed consent was obtained from all participants. All participants had normal or correct-to-normal vision and normal hearing.

#### Materials

A total of 32 sets of sentences were constructed with the verbs and names selected from the pre-test. All sentences consisted of two clauses, i.e., C1 and C2. C1 always began with a temporal adverb, followed by the structure *NP1 V NP2*, where the verb was either biased to NP1 or NP2. C2 was the causal clause led by *yinwei* “because,” followed by an ambiguous Ta and a six-character predicate that helped resolve the co-reference of Ta. The information in C2 disambiguated the pronoun to either the verb-consistent NP or the verb-inconsistent NP. See Table 2 for an example set of stimuli. All materials are available in the Supplementary Materials.

**TABLE 2:** Example stimuli in Experiments 1 and 3.

Condition	Example							
(a) NP1-biased, verb-consistent	上午	春丽	震惊了	小静，	(好几次)	因为	Ta	破了游泳纪录
	shangwu	Chunli	zhenjingle	Xiaojing	(haojici)	yinwei	Ta	poleyouyongjilu
	morning	Chunli	surprise-le	Xiaojing	(many times)	because	Ta	break the swimming record
	‘Chunli surprised Xiaojing in the morning (for many times), because Ta broke the swimming record.’							
(b) NP1-biased, verb-inconsistent	上午	春丽	震惊了	小静，	(好几次)	因为	Ta	一直期待很低。
	shangwu	Chunli	zhenjingle	Xiaojing	(haojici)	yinwei	Ta	yizhiqidaihendi
	morning	Chunli	surprise-le	Xiaojing	(many times)	because	Ta	always has a low expectation
	‘Chunli surprised Xiaojing in the morning (for many times), because Ta has always had a low expectation.’							
(c) NP2-biased, verb-consistent	上午	春丽	安慰了	小静，		因为	Ta	失去参赛资格。
	shangwu	Chunli	anweile	Xiaojing		yinwei	Ta	shiqucansaizige
	morning	Chunli	comfort-le	Xiaojing		because	Ta	lost the eligibility
	‘Chunli comforted Xiaojing in the morning, because Ta lost the eligibility for the competition.’							
(d) NP2-biased, verb-inconsistent	上午	春丽	安慰了	小静，		因为	Ta	感到特别同情。
	shangwu	Chunli	anweile	Xiaojing		yinwei	Ta	gandaotebietongqing
	morning	Chunli	comfort-le	Xiaojing		because	Ta	feel very sympathetic
	‘Chunli comforted Xiaojing in the morning, because Ta felt very sympathetic.’							

*Experiment 3 shared the NP1-biased conditions with Experiment 1 but with the inclusion of texts in parentheses.*

A number of factors were controlled for the six-character predicates in C2. First, we extracted the log-frequencies of each word/segment in C2 from the SUBTLEX-CH corpus (Cai and Brysbaert, 2010). The mean log-frequency of all segments in a predicate were calculated and treated as the log-frequency of that predicate. Second, the syntactic complexity of C2 and the semantic role of Ta in C2 were manually coded by a trained Chinese linguist. In terms of syntactic complexity, predicates with more than two components [e.g., noun phrase (NP), adjective phrase (AP), verb phrase (VP)] were treated as complex structures and coded as “1,” and those with two or less components were treated as simple structures and coded as “0.” As for semantic roles, predicates where Ta was agent were coded as “1” and those where Ta was non-agent were coded as “0.” The descriptive data of these factors are provided in Table 3, with linear/logistic regression results showing no significant differences across conditions in terms of each variable (*p*s > 0.07).

**TABLE 3:** Parameters of predicates in C2.

Condition	Experiment 1			Experiment 2		
	Log-frequency	Syntactic complexity	Semantic role	Log-frequency	Syntactic complexity	Semantic role
a	3.47 (0.96)	0.75 (0.44)	0.25 (0.44)	3.72 (0.63)	0.84 (0.37)	0.34 (0.48)
b	3.61 (0.81)	0.81 (0.40)	0.22 (0.42)	3.55 (0.63)	0.78 (0.42)	0.25 (0.44)
c	3.38 (0.81)	0.69 (0.47)	0.34 (0.48)	3.89 (0.79)	0.84 (0.37)	0.28 (0.46)
d	3.36 (0.81)	0.88 (0.34)	0.16 (0.37)	3.59 (0.93)	0.81 (0.40)	0.19 (0.40)

In order to examine whether the disambiguating predicates we constructed can successfully resolve the pronoun, we conducted a norming test by asking 12 native speakers (3 men, 9 women, mean age 20.42 years, range 18–23 years) to read the sentences that were to be used in the real experiment and judge which person Ta refers to. None of this group of participants participated in any other experiments reported in this study. We divided the materials into four Latin square lists and each participant was assigned to one of the four lists. The accuracy was above 92% for all conditions with logistic regression results showing no significant differences among them (*p*s > 0.2), suggesting that the pronouns in the constructed materials were able to be resolved as expected^5^.

The 32 sets of items, together with the materials of our other study that had a similar design (not reported here), were divided into eight counter-balanced lists for the online processing experiment. Each list consisted of four sets (altogether 16 trials) of critical items of the current study. In addition to the 16 critical trials of the current study and the 16 trials from our other study, we added 36 two-clause fillers with various syntactic structures to each list, resulting in a full list of 68 sentences for each participant. Each critical sentence was followed by a comprehension question that was identical across conditions, i.e., “Who do you think Ta in the last sentence refers to?”, with NP1 and NP2 as its potential answers. All fillers also came with a comprehension question with two potential answers, one of which was the correct answer. Part of the filler questions were the same as critical items and the others were targeted at other contents in the sentence.

All sentences were recorded by a woman native speaker at the frequency of 44,100 Hz in a quiet room using Praat (Boersma, 2001). The two clauses in a sentence were recorded as one audio file so as to maintain the natural intonation of the sentence. There was a natural stop between two clauses for all experimental items and fillers, which varied between 190 and 620 ms. 95% of the experimental items had an interval between 300 and 500 ms.

#### Procedure

Participants were instructed to sit in a quiet room and randomly assigned to one of the eight lists. Before each trial, there was a fixation “+” on the screen. Participants pressed the SPACE bar on the keyboard to proceed to the next page, on which they looked at a blank screen while listening to the audio of the whole sentence over headphones. After the audio stopped, a comprehension question appeared visually on the screen with its two potential answers. The position of the correct answer was balanced across all trials. Participants were instructed to use F or J on the keyboard to make the choice as fast and as accurately as possible. Participants’ decision and their response time (i.e., the time from the onset of the comprehension question till participants’ press of the key) were automatically recorded by the program. All trials were presented pseudo-randomly using E-Prime 3 (Psychology Software Tools, Pittsburgh, PA, United States). Participants went through four practice trials before the real experiment so as to get familiar with the experimental procedure. The whole experiment took about 15 min to complete.

#### Data Analysis

Only the trials where participants successfully reached the expected referent of the pronoun were analyzed. Linear mixed effects models were fit to the response time data using lme4 package version 1.1–27.1 (Bates et al., 2015) in R version 4.1 (R Core Team, 2021). The lmerTest package version 3.1–3 (Kuznetsova et al., 2017) in R was used for demonstrating the *p*-value. The fixed effects were *consistency* and *verb*, both of which were manually sum coded (verb-consistent as –0.5 and verb-inconsistent as +0.5; NP1-biased verb as –0.5 and NP2-biased verb as +0.5). To more precisely observe how verb bias strength modulates online pronoun resolution, we included the referential probability of the bias-congruent referent for each verb as a covariate called *strength*, which was scaled and centered for NP1-biased verbs and NP2-biased verbs respectively using *scale()* function in R. The response times were log-transformed to stabilize variance and achieve approximately normal residuals (Box and Cox, 1964). As for random effects, since each set of item contains a particular verb that comes with a specific bias strength, which has already been included as a covariate in our model, only subject was included as a random effect. Each model was initially built with maximum random intercepts and random slopes for subjects, and the random slope was eliminated stepwise if the model failed to converge. *Post hoc* tests were conducted following significant interactions with Bonferroni correction.

### Results

**FIGURE 1:**
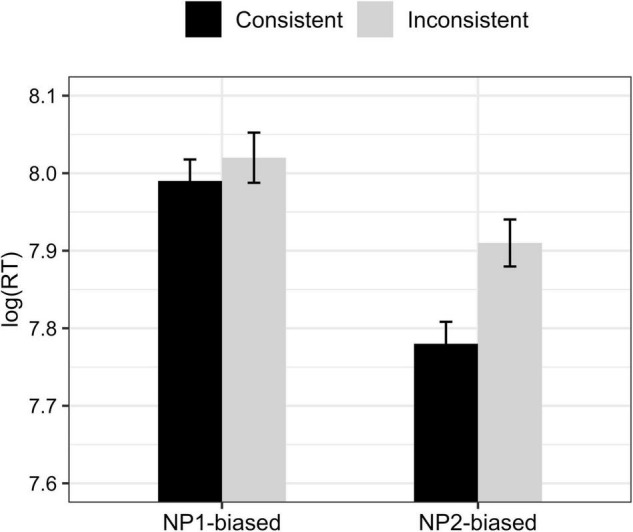
Mean log-transformed response times in each condition of Experiment 1. Error bars represent one standard error.

The overall comprehension accuracy was 93.69% (*SD* = 0.24) for all experimental trials and fillers, suggesting that participants generally performed well in the task. Experimental trials to which participants answered incorrectly were excluded for analysis (accounting for 14.3% of the total experimental trials). For the remaining trials, response times were replaced by NAs for values more than two standard deviations above or below the mean (accounting for 3% of the remaining observations). The mean response times of each condition are shown in Figure 1, and statistical results are given in Table 4.

**TABLE 4:** Model output of Experiment 1.

	Estimate	Std. Error	*t* value	*p*
(Intercept)	7.93057	0.03129	253.463	<2e-16***
consistency	0.09382	0.02496	3.759	0.000182***
verb	–0.15458	0.02510	–6.157	1.1e-09***
strength	0.01981	0.01369	1.447	0.152073
consistency:verb	0.09311	0.04992	1.865	0.062457
consistency:strength	0.09874	0.02527	3.907	0.000100***
verb:strength	–0.04436	0.02574	–1.723	0.085186
consistency:verb:strength	–0.01386	0.05058	–0.274	0.784078

***p < 0.001. Formula in R: logRT ∼ consistency * verb * strength + (1 + strength | Subject).

We found a main effect of *verb* showing that NP2-biased sentences were processed with significantly less time than NP1-biased sentences, which replicated the results of previous studies on other language than Chinese (e.g., Dery and Bittner, 2016). In addition, we found a main effect of *consistency* showing that verb-inconsistent sentence took longer time to process than verb-consistent sentence and a consistency*strength interaction. *Post hoc* tests with Bonferroni correction showed that whereas verb-inconsistent sentences became significantly easier to process as the strength of verb bias decreases (β = 0.06, *t* = 2.72, adjusted *p* < 0.05), verb bias strength had no effect on the processing time of verb-consistent sentences (adjusted *p* > 0.1), which we suggest was due to a ceiling effect. That is, participants were always very fast to respond to verb-consistent sentences such that their response time to verb-consistent sentences could not be significantly faster as the verb bias becomes stronger. The effect of verb bias strength has reached a top limit in affecting/facilitating participants’ processing of the verb-consistent sentences.

### Discussion

In this experiment we adopted the structure [_*C1*_ *NP1 V NP2*] [_*C2*_ *because Pro…*] and included both NP1-biased verbs and NP2-biased verbs and manipulated the disambiguating information in C2 as either verb-consistent or verb-inconsistent. Results showed that verb-inconsistent sentences need more time to process than verb-consistent sentences, which replicated the results of previous studies that verb bias direction plays an important role in online pronoun resolution (e.g., Caramazza et al., 1977; Vonk, 1985; Guerry et al., 2006). Importantly, with the inclusion of the verb bias strength in our model, we found an interaction between the consistency of the disambiguating information in C2 and the verb bias strength in C1 such that whereas verb-consistent sentences remained easy to process regardless of the strength of verb bias, which we suggest was due to a ceiling effect, verb-inconsistent sentences became significantly easier to process as verb bias strength decreases. This experiment provided a more precise picture that implicit causality is a continuum and that the subtle difference in the strength of verb bias has an effect on online pronoun resolution. Using Chinese as its materials, Experiment 1 provided evidence that implicit causality is valid across languages and cultures (Hartshorne et al., 2013).

## Experiment 2

Experiment 2 aimed at investigating whether and how the verb-based implicit causality in C1 of backward concession affects pronoun resolution in the subsequent C2. Different from backward causality that contains a real-world cause-consequence relationship shared in both the speaker’s and the hearer’s common knowledge, backward concession is an intersubjective relation in which the two clauses are in an indirect relationship. We therefore expect the implicit causality to be less effective on pronoun resolution in backward concession.

### Methods

#### Participants

In this experiment, 24 (8 men, 16 women, mean age 21.17 years, range 18–25 years) native speakers of Mandarin Chinese were paid to participate. None of them took part in the pre-test or Experiment 1. All participants had normal or correct-to-normal vision and normal hearing and gave their informed consent.

#### Materials

Following the same structure as in Experiment 1, 32 sets of items were created except that we changed the connective from *yinwei* “because” to *suiran* “although.” Similar to Experiment 1, we included both NP1-biased verbs and NP2-biased verbs, and the information in C2 disambiguated the pronoun to either the verb-consistent NP or the verb-inconsistent NP. The parameters of the six-character predicate in C2 are given in Table 3. See Table 5 for an example set of stimuli.

**TABLE 5:** Example stimuli in Experiments 2 and 3.

Condition	Example							
(a) NP1-biased, verb-consistent	上午	春丽	震惊了	小静，	(好几次)	虽然	Ta	只是正常发挥。
	shangwu	Chunli	zhenjingle	Xiaojing	(haojici)	suiran	Ta	zhishizhengchangfahui
	morning	Chunli	surprise-le	Xiaojing	(many times)	although	Ta	just perform normally
	‘Chunli surprised Xiaojing in the morning (for many times), although Ta just performed normally.’							
(b) NP1-biased, verb-inconsistent	上午	春丽	震惊了	小静，	(好几次)	虽然	Ta	早有心理准备。
	shangwu	Chunli	zhenjingle	Xiaojing	(haojici)	suiran	Ta	zaoyouxinlizhunbei
	morning	Chunli	surprise-le	Xiaojing	(many times)	although	Ta	had been mentally prepared
	‘Chunli surprised Xiaojing in the morning (for many times), although Ta had been mentally prepared.’							
(c) NP2-biased, verb-consistent	上午	春丽	安慰了	小静，		虽然	Ta	并不是很领情。
	shangwu	Chunli	anweile	Xiaojing		suiran	Ta	bingbushihenlingqing
	morning	Chunli	comfort-le	Xiaojing		although	Ta	not really appreciate it
	‘Chunli comforted Xiaojing in the morning, although Ta did not really appreciate it.’							
(d) NP2-biased, verb-inconsistent	上午	春丽	安慰了	小静，		虽然	Ta	只是进行敷衍。
	shangwu	Chunli	anweile	Xiaojing		suiran	Ta	zhishijinxingfuyan
	morning	Chunli	comfort-le	Xiaojing		although	Ta	just being perfunctory
	‘Chunli comforted Xiaojing in the morning, although Ta was just being perfunctory.’							

*Experiment 3 shared the NP1-biased conditions with Experiment 2 but with the inclusion of texts in parentheses.*

The 32 sets of materials, each consisting of four conditions, were divided into four counter-balanced lists in a Latin square design. Each list consisted of 32 critical items (eight per condition). Similar to Experiment 1, the materials were normed by a separate group of 12 native speakers (4 men, 8 women, mean age 24.83 years, range 22–32 years) who read the sentences to be used in the real experiment and judged which person Ta refers to. Each participant was assigned to one of the four lists and did not participant in the any other experiment reported in this study. The accuracy was above 94% for all conditions with logistic regression results showing no significant differences among them (*p*s > 0.9), suggesting that the pronouns in the constructed materials were able to be resolved as expected. We then added 36 two-clause fillers with various syntactic structures to each list, altogether forming a list of 68 sentences for each participant. The comprehension questions following critical items and fillers followed the same format as described in Experiment 1.

All sentences were recorded in the same way as in Experiment 1 except that the two clauses in critical sentences were recorded as separate audio files. Since the two NP1-biased conditions had identical C1, we only recorded C1 once and applied it to both NP1-biased verb-consistent and NP1-biased verb-inconsistent conditions. The same procedure was applied for the two NP2-biased conditions. Fillers were recorded as a whole sentence. The natural stop between the two clauses in fillers was between 180 and 360 ms.

#### Procedure and Data Analysis

This experiment followed the same procedure as described in Experiment 1 except that the critical sentences were played clause by clause with a fixed inter-stimulus interval (ISI) of 200 ms. The two clauses were played automatically by the program. Fillers were played in a whole sentence, but there was a natural stop between two clauses that varied between 180 and 360 ms. Like Experiment 1, participants looked at a blank screen while listening to the audios. The whole experiment took about 15 min to complete. Data analysis followed the same process with that of Experiment 1.

### Results

**FIGURE 2:**
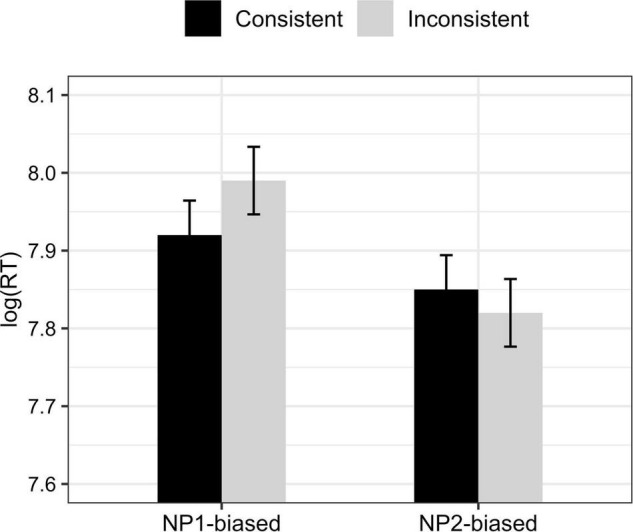
Mean log-transformed response times in each condition of Experiment 2. Error bars represent one standard error.

The overall comprehension accuracy was 88.24% (*SD* = 0.32) for all experimental trials and fillers, suggesting that in generally participants performed well in the task. Experimental trials that were answered incorrectly were excluded for analysis (accounting for 20.8% of the total experimental trials). For the remaining trials, we replaced response times with NAs for values more than two standard deviations above or below the mean (accounting for 4% of the remaining observations). The mean response times of each condition are shown in Figure 2.

Statistical results only showed a main effect of *verb*, with less time being spent in processing NP2-biased sentences than processing NP1-biased sentences. The model output is given in Table 6.

**TABLE 6:** Model output of Experiment 2.

	Estimate	Standard error	*t-*value	*p*
(Intercept)	7.89572	0.04062	194.396	<2e-16***
consistency	0.03667	0.04153	0.883	0.37767
verb	–0.11593	0.04146	–2.797	0.00535**
strength	0.01554	0.02143	0.725	0.47801
consistency:verb	–0.12661	0.08576	–1.476	0.14038
consistency:strength	–0.03246	0.04253	–0.763	0.44568
verb:strength	–0.06324	0.04266	–1.483	0.13876
consistency:verb:strength	0.14662	0.08521	1.721	0.08592

***p < 0.001, **p < 0.01. Formula in R: logRT ∼ consistency * verb * strength + (1 + strength | Subject).

### Discussion

In Experiment 2 we included both NP1-biased verbs and NP2-biased verbs and manipulated the disambiguating information in C2 of a backward concessive relation as either verb-consistent or verb-inconsistent. Similar to Experiment 1, we found a main effect of *verb* showing a processing advantage of NP2-biased conditions. The results extend previous studies that found a facilitated processing of NP2-biasd conditions in causal relations (e.g., Dery and Bittner, 2016) and suggest a broader application of the NP2-biased advantage to other discourse relations like backward concession. More importantly, our results showed no difference in the time taken to comprehend the verb-consistent condition and the verb-inconsistent condition, suggesting that comprehenders can quickly accommodate the disambiguating information in C2 to correctly resolve the pronoun in backward concession no matter which antecedent the pronoun is disambiguated to. The results suggest that verb-based implicit causality in C1 has limited effect on participants’ online resolution of the pronoun in C2 in backward concession, which we attribute to the fact that backward concession by default is an intersubjective relation where the concessive clause is a retreating statement in an indirect relationship with C1.

While Experiment 2 showed a distinct effect of implicit causality in backward concession from causality, this difference could have been caused by the different groups of participants in Experiments 1 and 2. To compare concession and causality more directly, we crossed the two discourse relations in a within-subject design and conducted Experiment 3.

## Experiment 3

Experiment 3 aimed at providing more direct evidence for the effect of implicit causality in the two closely related discourse relations, i.e., concession and causality. To reduce the complexity of the design, only NP1-biased verbs were included in this experiment. In a 2 × 2 within-subject design, we crossed discourse relation (causality vs. concession) and the consistency of the disambiguating information in C2 (verb-consistent vs. verb-inconsistent).

### Methods

#### Participants

In Experiment 3, 24 (8 men, 16 women, mean age 21.33 years, range 18–27 years) native speakers of Mandarin Chinese were paid to participate. None of them took part in the pre-test or previous experiments. All participants gave their informed consent and had normal or correct-to-normal vision and normal hearing.

#### Materials

The 32 sets of NP1-biased conditions from Experiments 1 and 2 were adopted. As a result, C2 in this experiment was led by either *yinwei* “because” or *suiran* “although,” and the information in C2 disambiguated the pronoun to either the verb-consistent NP or the verb-inconsistent NP, resulting in four conditions in total. A slight modification in this experiment was that we added an adverbial phrase in the end of C1 (i.e., after NP2) that emphasizes the action denoted by the verb (Lü, 1942; Chao, 1968; Zhu, 1982). With this modification, we expect to strengthen the bias carried by the NP1-verb and bring the effect of verb bias direction more salient. The temporal adverbs before NP1 were changed accordingly if necessary. Example stimuli in each condition can be found in Tables 2, 5.

The 32 sets of materials, each containing four conditions, were divided into four lists in a Latin square design. Each list consisted of 32 critical items (eight per condition). Like previous experiments, the materials were normed by a separate group of 12 participants (four men, eight women, mean age 25.08 years, range 21–37 years) who did not participant in any other experiment reported in this study. Each subject was assigned a Latin square list and asked to read the whole sentences that were to be used in the real experiment and judge which person Ta refers to. The accuracy was above 94% for all conditions with logistic regression results showing no differences among them (*p*s > 0.9), suggesting that the pronouns in the constructed materials were able to be resolved as expected. Similar to previous experiments, we added 36 two-clause fillers with various syntactic structures to each list and resulted in a full list of 68 trials for each subject. Each trial was followed by a comprehension question that was constructed in the same format as described in Experiment 1.

All sentences were recorded by a woman native speaker at the frequency of 44,100 Hz in a quiet room using Praat (Boersma, 2001). Similar to Experiment 2, the two clauses in critical sentences were recorded as separate audio files. Since C1 was identical across all four conditions, we only recorded C1 once and applied it to all conditions. Fillers were recorded as a whole sentence. The natural stop between the two clauses in fillers was between 220 and 490 ms.

#### Procedure and Data Analysis

The procedure was the same as that of Experiment 2. With respect to data analysis, this experiment had a new sum-coded fixed effect *relation* (causality as –0.5 and concession as +0.5) in addition to previously introduced fixed effects *consistency* and *strength*. The data were analyzed following the same steps with previous experiments.

### Results

**FIGURE 3:**
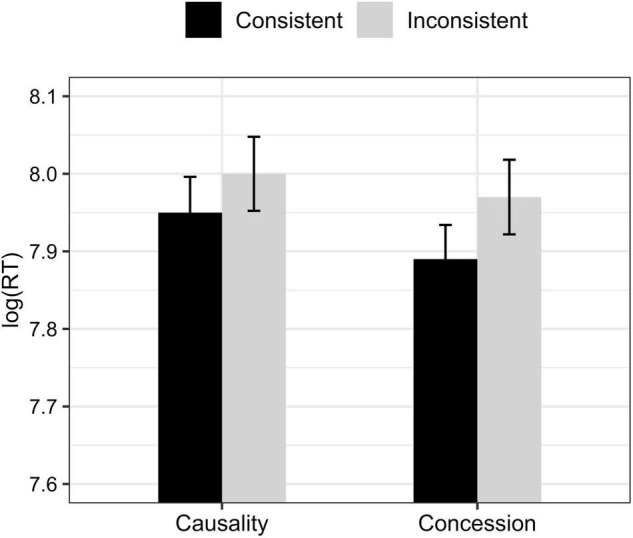
Mean log-transformed response times in each condition of Experiment 3. Error bars represent one standard error.

The overall comprehension accuracy was 85.05% (*SD* = 0.36) for all experimental trials and fillers, suggesting that participants generally performed well in the task. Experimental trials that participants answered incorrectly were excluded for analysis (accounting for 19.3% of the total experimental trials). For the remaining trials, we replaced response times with NAs for values more than two standard deviations above or below the mean (accounting for 5% of the remaining observations). The mean response times of each condition are shown in Figure 3, and statistical results are given in Table 7.

**TABLE 7:** Model output of Experiment 3.

	Estimate	Standard error	*t*-value	*p*
(Intercept)	7.95772	0.06373	124.871	<2e-16***
consistency	0.05521	0.04029	1.370	0.1829
strength	0.05183	0.02026	2.558	0.0108*
relation	–0.04357	0.03891	–1.120	0.2633
consistency:strength	0.08791	0.04044	2.174	0.0301*
consistency:relation	0.01567	0.07783	0.201	0.8405
strength:relation	0.00388	0.04047	0.096	0.9237
consistency:strength:relation	–0.18592	0.08166	–2.277	0.0232*

***p < 0.001, *p < 0.05. Formula in R: logRT ∼ consistency * strength * relation + (1 + consistency | Subject).

**FIGURE 4:**
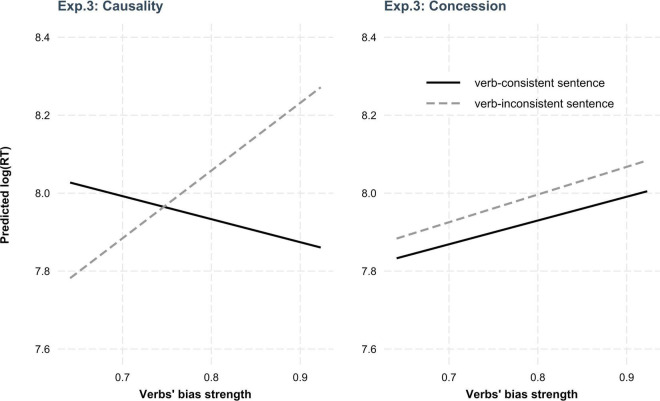
Plot illustrating the three-way interaction among *consistency*, *strength*, and *relation* in Experiment 3.

There was a main effect of *strength* showing that regardless of sentence consistency and discourse relation, more processing time was needed as verb bias strength increased. In addition, we found a two-way interaction between *consistency* and *strength*, suggesting that the effect of *strength* was in fact modulated by the effect of *consistency*. More importantly, there was a three-way interaction among *consistency*, *strength*, and *relation*, which further suggests that the two-way consistency*strength interaction was modulated by the effect of *relation*. In order to better understand the main effects and interactions, we spelled out the three-way interaction in Figure 4.

As can be seen from Figure 4, the effects of *consistency* and *strength* elicited different patterns in causality and concession. *Post hoc* tests with Bonferroni correction showed a significant interaction between *consistency* and *strength* in causality (β = 0.19, *t* = 3.03, adjusted *p* < 0.01). Further *post hoc* tests showed that whereas the processing of verb-consistent conditions was not affected by the strength of verb bias (adjusted *p* > 0.7), verb-inconsistent sentences need significantly less time to process as verb bias strength decreases (β = 0.14, *t* = 3.27, adjusted *p* < 0.01). In concession, on the other hand, we did not find any main effects nor interaction (adjusted *p*s > 0.09), suggesting that verb-consistent and verb-inconsistent conditions did not differ in terms of the time taken to arrive at the correct interpretation of the pronoun.

### Discussion

This experiment adopted a within-subject design with sentences like [_*C1*_ *NP1 V NP2*] [_*C2*_ *because/although Pro…*], where we manipulated discourse relation (concession vs. causality) and consistency of the disambiguating information in C2 (verb-consistent vs. verb-inconsistent). Results showed a consistency*strength interaction in causality but no main effects nor interactions in concession, which replicated the results of Experiment 1 (causality) and Experiment 2 (concession), respectively. To be specific, whereas the time taken to arrive at the correct interpretation of the pronoun in backward causality was highly modulated by the direction and strength of verb bias, in backward concession the implicit causality information carried by verb in C1 had very limited effects on the interpretation of the pronoun in C2, as no differences were found in the processing time of verb-consistent and verb-inconsistent conditions.

## General Discussion

This study sought to investigate the effect of verb-based implicit causality on pronoun resolution in intersubjective discourse relations like backward concession. Experiment 1 provided a first glimpse into how implicit causality affects online pronoun resolution using [_*C1*_ *NP1 V NP2*] [_*C2*_ *because Pro…*], where we included both NP1-biased verbs and NP2-biased verbs and manipulated the disambiguating information in C2 as either consistent or inconsistent with the verb bias. We found an interaction between the consistency of the sentence and the strength of verb bias with *post hoc* testing showing that verb-inconsistent sentences became significantly easier to process as verb bias strength decreases. This result provides a more comprehensive picture of implicit causality such that verbs differ in terms of their bias strength in affecting online pronoun resolution. In Experiment 2, we used the same verbs as in Experiment 1 but replaced the connective *because* with *although*. Results showed no differences in the time taken to arrive at the correct interpretation of the pronoun between the verb-consistent condition and the verb-inconsistent condition, suggesting that verb-based implicit causality in C1 of backward concession has limited effects on participants’ online resolution of the pronoun in the subsequent C2. Finally, in Experiment 3, we crossed concession and causality in a within-subject design and replicated the main findings of Experiments 1 and 2.

In this study, we found a main effect of consistency in the baseline causal relation showing that verb-inconsistent sentences were processed with more time than verb-consistent sentences (Experiments 1 and 3). The results replicate previous finding that comprehenders make use of the implicit causality information in the verb to resolve the pronoun during online comprehension (Caramazza et al., 1977; Vonk, 1985; Garnham et al., 1996; Stewart et al., 2000; Garnham, 2001; Guerry et al., 2006; Koornneef and van Berkum, 2006; van Berkum et al., 2007; Pyykkönen and Järvikivi, 2010; Cozijn et al., 2011; Järvikivi et al., 2017). By including the strength of the verb bias in our statistical model, the present study provides a more precise picture of the implicit causality effect such that as verb bias strength decreases, verb-inconsistent sentences became significantly easier to process. When the verb does not strongly bias the pronoun (i.e., the potential cause of the event) to NP1, it leaves more chance of interpreting the pronoun as the verb-inconsistent NP2, and therefore integrating the disambiguating information to NP2 is not cognitively demanding. However, when the verb semantics becomes stronger in biasing the pronoun to NP1, it requires more cognitive efforts to integrate the verb-inconsistent information to the preceding main clause to arrive at the correct interpretation of the sentence. Using Chinese as its materials, the present study speaks for the influence of implicit causality as a cross-linguistic phenomenon (Hartshorne et al., 2013).

In Experiments 2 and 3 where backward concession was investigated, we found no main effect of consistency nor any interaction on participants’ response time, suggesting that it requires equal cognitive efforts to comprehend all concessive conditions, regardless of whether it was NP1-biased or NP2-biased and whether the sentence was consistent or inconsistent with the verb bias. Different from the situation in backward causality, resolving the pronoun in backward concession is not affected by the direction of verb bias. Our results are consistent with previous studies that showed greater uncertainty upon encountering the pronoun in *although-*clause. While a number of studies found an immediate effect of implicit causality when comprehenders reach the pronoun in *because*-clause (Koornneef and van Berkum, 2006; van Berkum et al., 2007; Pyykkönen and Järvikivi, 2010; Cozijn et al., 2011; Järvikivi et al., 2017), Xu et al. (2019) found that by the time comprehenders encounter the pronoun in a concessive sentence like *Lily disappointed Nina, although she quit the business*, it remains unclear which antecedent in the previous discourse *she* refers to, which elicits a larger sustained negativity compared to other conditions where the two clauses are connected by *so*, *and*, or a full stop (see additional studies from Kehler, 2002; Kehler et al., 2008; Bott and Solstad, 2014). Xu et al.’s (2019) results suggest that participants’ interpretation of the pronoun in concession is to a less extent affected by the verb bias in C1 by the time participants reached the pronoun. In the current paradigm where participants resolved the pronoun after hearing the complete sentence, we found no differences in the time taken to successfully comprehend the verb-consistent information or the verb-inconsistent information, suggesting that the direction of verb bias in C1 in backward concession has limited effects on the interpretation of the ambiguous pronoun in C2 even *after* the disambiguating information has unfolded. Together, Xu et al. (2019) and our study suggest an indirect relationship between the two clauses in backward concession such that resolving the pronoun of C2 is independent from the implicit causality information carried by the verb in C1.

It is worth noting, however, that the lack of implicit causality effect in backward concession does not suggest participants resolved the pronoun in C2 without referring to any information in the preceding context. Participants still need to resort to C1 to fulfill the experimental task, i.e., to correctly identify the referent of the pronoun in discourse. Certain information in context, for instance, the semantic association between *appreciate* and *comfort* in *Chunli comforted Xiaojing in the morning, although Ta did not really appreciate it*, helped the comprehender to locate the potential referent of *Ta* to one of the antecedents in C1, which is, in this case, the patient *Xiaojing*. It is only the implicit causality information carried by the verb, i.e., the potential of the verb to bias the cause of the event to one of the antecedents, that participants were not sensitive to when they resolved the pronoun in backward concession, and this is because, as we have discussed, the two clauses in backward concession do not form a tight (negated) cause-consequence relationship but instead an indirect relationship where the subordinate concessive clause serves as a retreating statement to the argument claimed in the preceding main clause.

Our results further corroborate previous studies that suggest comprehenders’ high tolerance in processing concession. In Townsend and Bever’s (1982) and Morera et al.’s (2017) experiments, for instance, comprehenders were able to comprehend the subsequent C2 of a concessive relation regardless of which type of information was provided in C1. In a similar vein, with forward concession like *Although he has a talent for language, he does not like to learn English/driving*, Lyu et al. (2020) showed that the implausible sentence with *driving* was first considered as anomalous but later became acceptable. The authors proposed a perspective shift account such that comprehenders are able to quickly adopt the speaker’s point of view and accommodate whatever she claims later, even if it is ‘‘implausible’’ at first glance. In the present study, there was no difference in the time taken to integrate the verb-consistent or verb-inconsistent disambiguating information in C2 on the ‘‘correctly comprehended’’ trials, suggesting that as long as comprehenders accepted the disambiguating information in *although*-clause (i.e., were able to successfully integrate it to the discourse representation), they were ready to accommodate it no matter which antecedent it refers to. These studies altogether point to a distinct feature of concession. That is, comprehenders are able to quickly accommodate the speaker’s point of view when processing concession, no matter whether the relation is in a forward or a backward order^6^. In forward concession where the claim in the main clause was manipulated, comprehenders were able to accept the speaker’s claim in the main clause, even though it seems implausible at an initial stage (Lyu et al., 2020); in backward concession where the predicate of the subordinate clause was manipulated, hearers can quickly integrate the predicate to the preceding clause to resolve the ambiguous pronoun, no matter which antecedent it refers to (the present study).

Previous studies have shown that processing subjective causal relations like *She worked hard, because she passed the oral English test* is more difficult than processing objective relations like *She passed the oral English test, because she worked hard* (Traxler et al., 1997; Canestrelli et al., 2013; Kleijn et al., 2021), since the former involves a subjective (but implicit) speaker who expresses opinions, arguments, or attitudes but the latter is describing real-world cause-consequence facts. The present study extends the literature by suggesting a *perspective alignment* between the comprehender and the speaker during the online processing of (inter)subjective discourse relations. As has been discussed, the present study and Lyu et al. (2020) have suggested that processing concession involves comprehenders’ quick shift of perspective to the speaker. In addition, there are studies showing that certain subjectivity markers, such as epistemic stance markers *according to Peter*, can facilitate the processing of subjective causal relations like *According to Peter, she worked hard, because she passed the oral English test*. It is suggested that epistemic stance markers function as processing instructions that help readers evaluate how the argument in the subordinate causal clause (i.e., *because she passed the oral English test*) supports the claim in the preceding main clause (i.e., *She worked hard*) (Wei, 2018; Wei et al., 2021). In fact, during online processing, epistemic stance markers like *according to Peter* cue the comprehender of the protagonist of the sentence being processed. In other words, when processing a sentence like *According to Peter, she worked hard, because she passed the oral English test*, comprehenders interpret the subjective causal relation from Peter’s point of view, which makes the subjective argument – made by Peter rather than based on comprehenders’ own world knowledge – easier to process.

**FIGURE 5:**
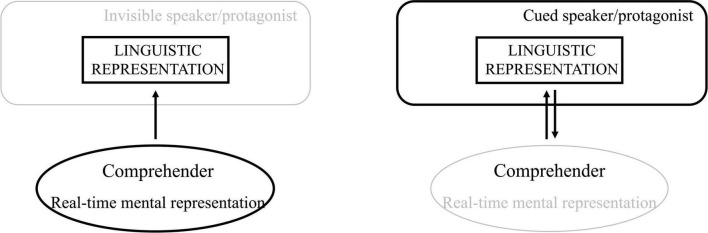
Perspective alignment between the comprehender and the speaker. The left panel illustrates the situation where comprehenders rely on their own world knowledge to build up the real-time mental representation (e.g., objective relations); the right panel illustrates the situation where the speaker/protagonist is made visible by certain linguistic cues (e.g., concessive connectives like *although*, epistemic stance markers like *according to Peter*) and the comprehenders’ ability to accommodate the speaker’s/protagonist’s attitude, argument, etc. when building up the real-time mental representation.

The above-mentioned studies altogether suggest an important ability of the online comprehender. As we show in Figure 5, during online processing, comprehenders not only rely on their own world knowledge to establish coherent discourse representations (left panel of Figure 5; e.g., McRae and Matsuki, 2009; Kuperberg et al., 2011; Cook and O’Brien, 2014) but are influenced by the subjective attitude of the speaker or the protagonist that is embedded, explicitly or implicitly, in the linguistic representation. Despite that the speaker’s subjective attitude brings about extra costs to online processing compared to an objective sentence, comprehenders are able to quickly accommodate the speaker’s or protagonist’s point of view in real time if there are cues for it (right panel of Figure 5; e.g., concessive connectives like *although* and epistemic stance markers like *according to Peter*).

As one of our reviewers pointed out, backward concession comes with a variety of other features than being intersubjective that could have led to the observed absent effect in the current study. For instance, a backward concessive relation might not be as frequently used as backward causality, and this could have led to an unfamiliarity to the participants, who therefore did not show any sensitivity to the verb-based implicit causality information during online processing. Given that the current study did not manipulate different dimensions/features of backward concession, other interpretations of the results remain possible and are worth testing in future experiments.

Before concluding, we acknowledge some limitations of this study and point out future directions. First, we did not distinguish different types of concession in the current study. While we grounded our study on the claim that concession in general is an intersubjective relation, there are still different degrees of (inter)subjectivity among different types of concession. How implicit causality plays a different role in content concession like (3a) and epistemic concession like (3b) is worth testing in future study. Second, the current study is restricted to the concessive connective *suiran* “although” in Chinese. Whether the results can be generalized to other connectives in other languages remain to be tested.

## Conclusion

This study investigated the effect of implicit causality on pronoun resolution in subjective discourse relations like backward concession. Experiment 1 examined implicit causality effect in backward causal relation, a typical discourse relation where verb-based implicit causality has been found to be effective. We showed that verb-inconsistent sentences were responded faster as the strength of verb bias decreases, which suggests that implicit causality is a continuum along which verbs differ in terms of their bias strength in affecting pronoun resolution. In Experiment 2, we tested the effect of implicit causality in backward concession and found no difference in comprehending verb-consistent and verb-inconsistent sentences, which suggests that participants were ready to accommodate the disambiguating information in C2 into the preceding clause to resolve the pronoun, no matter which antecedent it refers to. Experiment 3 replicated the key findings of the previous two experiments. We conclude by suggesting that certain linguistic cues (e.g., concessive connectives like *although*, epistemic stance markers like *according to Peter*) indicate a subjective perspective from the speaker or the protagonist and that comprehenders are able to quickly adopt the speaker’s or protagonist’s of view during online discourse processing.

## Data Availability Statement

The raw data supporting the conclusions of this article will be made available by the authors, without undue reservation.

## Ethics Statement

The studies involving human participants were reviewed and approved by Institutional Review Board, College of Foreign Languages, Zhejiang University of Technology. The patients/participants provided their written informed consent to participate in this study.

## Author Contributions

SL came up with the original research idea, conducted the experiment, ran statistics, and wrote and revised the manuscript. LW helped shape the idea and discussed the design with SL, revised and edited the manuscript. Both authors contributed to the article and approved the submitted version.

## Conflict of Interest

The authors declare that the research was conducted in the absence of any commercial or financial relationships that could be construed as a potential conflict of interest.

## Publisher’s Note

All claims expressed in this article are solely those of the authors and do not necessarily represent those of their affiliated organizations, or those of the publisher, the editors and the reviewers. Any product that may be evaluated in this article, or claim that may be made by its manufacturer, is not guaranteed or endorsed by the publisher.

## Footnotes

1
https://wj.qq.com

2 The pre-test results showed that Chinese NP2-biased verbs in general had a much higher bias-congruent referential probability than NP1-biased verbs. Rather than simply using the 32 highest NP1-biased verbs and the 32 highest NP2-biased verbs, we manually selected 32 verbs for each verb type and matched their referential probability. Within each verb type, the 32 verbs had different bias strengths, which, according to our prediction, will modulate the processing time.

3 A *t*-test showed that the selected 32 NP1-biased verbs and 32 NP2-biased verbs were statistically comparable to each other. As one of our reviewers pointed out, inferential statistics like *t*-test gives predictions at the population level. Here, we only used the *t*-test result to show that the mean referential probabilities of the selected samples do not significantly differ from each other, not necessarily that all Chinese NP1-biased verbs do not differ from all Chinese NP2-biased verbs.

4 As practiced in previous processing experiments, we kept the mean referential probability above 75% for both types of verbs but the range of the strength as low as 64% (e.g., Pyykkönen and Järvikivi, 2010; Järvikivi et al., 2017; Xu et al., 2019).

5 We acknowledge that due to the restricted length of the predicates, there were rare cases where participants might resolve the pronoun to the antecedent that did not match our expectations, especially in Experiment 2 where the intersubjective concessive relation was used. What we describe as “correct answer” in the remaining of the manuscript refers to trials where participants successfully reached the referent of the pronoun that matched our expectations, i.e., they reached bias-congruent interpretation of the pronoun in our verb-consistent conditions and bias-incongruent interpretations of the pronoun in our verb-inconsistent conditions. Only the response times of these “correctly answered” trials were analyzed.

6 As one of our reviewers pointed out, there could be an alternative interpretation from Grice’s theory of Conversational Maxims (Grice, 1989). According to the maxim of relation, the hearer expects to be provided with relevant information and, therefore, tries to make sense out of the incoming sentence.
